# One-stage Revision in a *Brucella* Prosthetic Hip Joint Infection with Late Presentation: A Case Report

**DOI:** 10.1055/s-0044-1787547

**Published:** 2024-12-27

**Authors:** Sandeep Gupta, Anmol Sharma, Jagseer Singh, Jatin Aggarwal

**Affiliations:** 1 Departamento de Ortopedia, Fortis Hospital, Mohali, Punjab, Índia

**Keywords:** arthroplasty, replacement, hip, bone cements, *Brucella*, hip, prosthesis-related infections

## Abstract

A 69-year-old female patient, who had been operated on 20 years ago (unipolar hip prosthesis), presented with a complaint of pain in the thigh and a limp with onset 1 year before. An X ray revealed stem subsidence and varus collapse. One-stage revision hip replacement was performed in view of poor cardiac status, and *Brucella melitensis* grew in the tissue culture. Oral doxycycline and rifampicin were administered for six weeks. The patient remained asymptomatic until the last follow up. Prosthetic joint infection by *B. melitensis* should be considered in a late onset, insidious presentation in an endemic country. One exchange arthroplasty with the administration of systemic antibiotics resulted in a good outcome.

## Introduction

Brucellosis is a zoonosis caused by Gram-negative bacteria of the genus *Brucella* , which is most commonly found in Mediterranean countries, Central Asia, Middle Eastern countries, and South America. Infection to humans is transmitted through contact with animals or contaminated animal products. 1 Osteoarticular infection is the most common complication of brucellosis (with rates ranging from 10% to 85%), and it causes large-joint infective arthritis, spondylitis, bursitis, tenosynovitis, and osteomyelitis. 2 Prosthetic joint infection (PJI) is a rare and less reported complication of brucellosis. 3 4 We herein describe a case of silent *Brucella* infection with an unusual presentation in a 20-year-old prosthetic hip; we also describe its management and outcome, and review the literature available on PJI with *Brucella* infection. The present is the first case of *Brucella* PJI reported in Southeast Asia, and the one with the longest time until onset of the infection after the primary surgery, according to the literature.

## Case Report

**Fig. 1:**
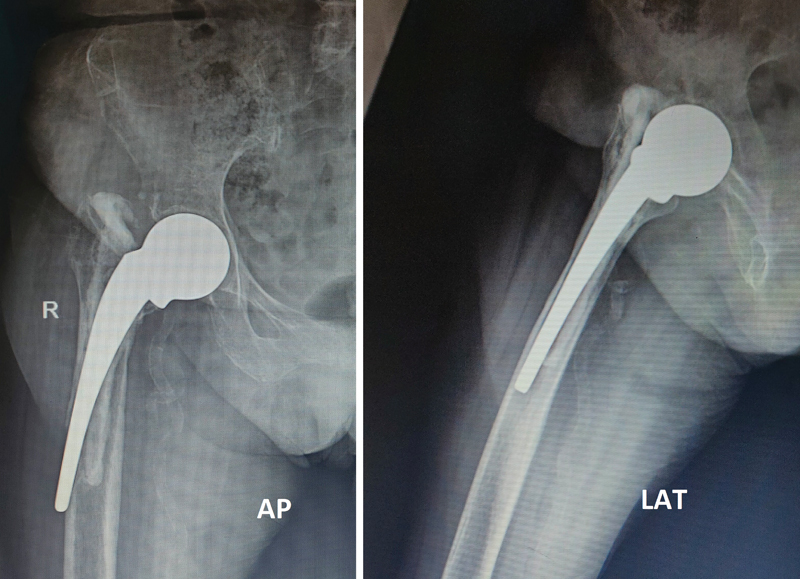
Preoperative radiograph on anteroposterior (AP) and lateral views.

A 69-year-old female patient operated on 20 years ago with a unipolar partial hip prosthesis for a displaced fracture of neck of femur presented with a complaint of pain in the upper thigh upon weight bearing with onset 3 months before and a limp with onset 1 year before. She had no history of recent falls, minor trauma or fever. The patient was a known diabetic and was a dairy farmer by occupation. Upon examination, the affected limb was shortened 2 cm, and all movements of the hip, especially rotations, were painful. The local examination was unremarkable. There was no history suggestive of local or systemic infection in the recent past or soon after the primary surgery. Radiographs revealed an old cemented monoblock unipolar prosthesis in situ with stem subsidence, varus collapse, and lateral cortex perforation ( Fig. 1 ). The blood workup revealed normal total and differential white cell counts, C-reactive protein (CRP) levels, and erythrocyte sedimentation rate (ESR). A single -tage revision hip replacement was planned due to the poor cardiac status of the patient.

**Fig. 2:**
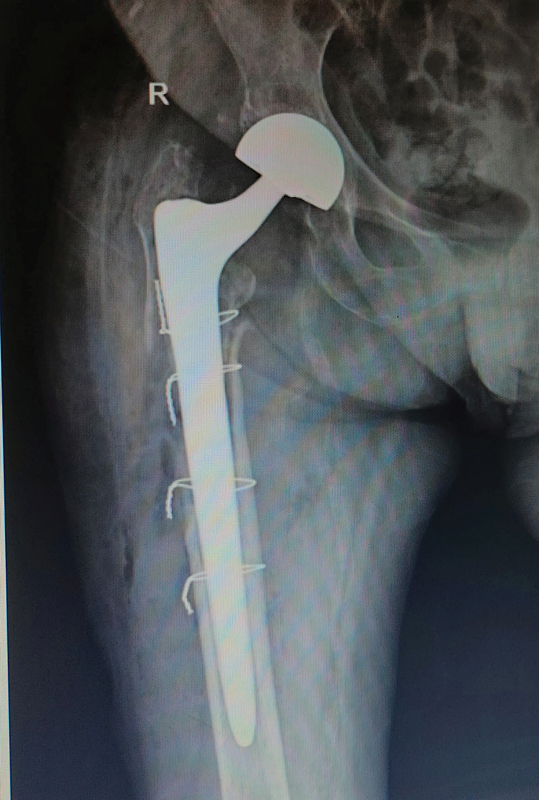
Immediate postoperative X-ray on AP view.

The affected hip was exposed using a modified Gibson approach. The implant was found slightly loose and, to the authors' surprise, there was a layer of whitish caseous material around the bone implant junction at the trochanteric level, which raised the suspicion of tubecular or a similar infection. An extended trochanteric osteotomy (ETO) was performed up to the level of the lateral cortex perforation of the stem tip, and the implant and cement were removed. Tissue samples were taken from five sites around the hip. An intraoperative leukocyte esterase test was negative. The wound was washed with hydrogen peroxide, 3 liters of 0.3% betadine saline, and closed temporarily. After repainting and draping, the wound was washed again with 3 liters of saline and prophylactic cerclage wiring was performed 2 cm distal to the perforation. A long uncemented Wagner-type stem measuring 20 mm x 240 mm was inserted. In view of the preserved acetabular cartilage, absence of any groin pain, persistent request from the anesthetist to finish the case within the least possible time, and blood loss due to the poor cardiac status of the patient, bipolar hemiarthroplasty was performed and the ETO was fixed using three cerclage wires ( Fig. 2 ). The wound was smeared with 2 gm of vancomycin powder around the hip after closure of the capsule.

Postoperatively, systemic cefuroxime was administered to the patient for five days. The postoperative period was eventful. Tissue culture showed growth of *Brucella melitensis* . After consultation with the infectious disease specialist, oral doxycycline and rifampicin were administered to the patient for six weeks. The patient remained asymptomatic thereafter, and no signs of local infection were observed until the last follow-up at 1 year.

## Review of Literature

The PubMed, PubMed Central (PMC), and Scopus databases were searched using the keywords *brucella* , *prosthetic joint injection* and *septic hip* . All results were screened for studies related to PJI due to *Brucella* , and the references of these studies were also evaluated. We found 30 articles on *Brucella* PJI which included 36 patients, 13 of whom were cases of hip PJI. Most of the patients were from Southern Europe and the Middle East. In total, 9 out of 13 (69.2%) patients had a history of contact with farm animals or consumption of unpasteurized dairy products. Infection after travel to endemic countries was reported by 5 patients out of 36 (13.89%). The mean time from prosthesis placement until the onset of infection was of 51 months. Most commonly, the symptoms were localized to the affected hip and thigh only (11 patients; 84.6%) while 1 (7.7%) patient presented systemic symptoms alone, and 1 (7.7%) presented both local and systemic symptoms. Implant loosening was observed in 9 (69.2%) patients on radiographs. The most common method of treatment was 2-stage revision hip replacement (7 patients; 53.8%). The outcome was reported to be good in all cases.

## Discussion

The diagnosis of *Brucella* PJI, especially in a non-endemic region, is a challenge, due to its rarity and usually insidious presentation. 5 In the case herein reported, the time between the placement of the prosthesis and the onset of infection was of 20 years, which was the longest ever documented to date. Moreover, our patient presented mild local symptoms without any systemic signs. In the literature, 6 7 two patients with *Brucella* PJI had a history of systemic brucellosis (one and six years after the arthroplasty respectively) before developing local syptoms. This becomes an important point in the history to diagnose *Brucella* PJI. Also, history of contact with farm animals or consumption of unpasteurized dairy products is important to elucidate. Serological tests for *Brucella* antibodies are a safe and reliable method of establishing a preoperative diagnosis, 8 but intraoperative tissue culture remains the most reliable method. There are no specific treatment guidelines due to the lack of large-scale, randomized, prospective studies with successful outcomes reported after one- and two-stage exchanges. Patients without any implant loosening and mild local symptoms have been treated with debridement alone or even conservatively, with good outcomes. Antimicrobial treatment usually consists of doxycycline and rifampicin with or without aminoglycoside, and the duration of the treatment ranges from 6 weeks to 2 years, depending on the subsidence of symptoms. 9 10

Additional prospective/multicenter studies that include samples with a wide range of ethnic backgrounds will help in the establishment of guidelines and appropriate treatment protocols.
