# Supplementary material for: One-stage Revision in a Brucella Prosthetic Hip Joint Infection with Late Presentation: A Case Report
Source: Rev Bras Ortop (Sao Paulo). 2024 Dec 27;59(Suppl 2):e255–8. doi: 10.1055/s-0044-1787547 (PMC11679613; doi:10.1055/s-0044-1787547)
Supplement: Related texts

# Research article: Revisão em um estágio de uma infecção protética da articulação do quadril por *Brucella* com apresentação tardia: Um relato de caso

**Keywords:** artroplastia de quadril, *Brucella*, cimentos ósseos, infecções relacionadas a prótese, quadril

## Abstract

Uma paciente do sexo feminino, de 69 anos, operada havia 20 anos (prótese unipolar de quadril), apresentou queixa de dor na coxa e claudicação havia 1 ano. A radiografia revelou subsidência da haste e colapso em varo. A artroplastia do quadril de revisão em um estágio foi realizada devido ao mau estado cardíaco da paciente, e a cultura de tecidos revelou a presença de *Brucella melitensis* . Doxiciclina e rifampicina foram administradas por via oral por seis semanas. A paciente permaneceu assintomática até o último acompanhamento. A infecção da prótese articular por *B. melitensis* deve ser considerada em casos de apresentação tardia e insidiosa em um país endêmico. A artroplastia de revisão e a administração de antibióticos sistêmicos levaram a um bom desfecho.

## Introdução

A brucelose é uma zoonose causada por bactérias Gram-negativas do gênero *Brucella* , que são mais comuns em países mediterrâneos, na Ásia Central, nos países do Oriente Médio e na América do Sul. A infecção humana é transmitida por meio do contato com animais ou produtos de origem animal contaminados. 1 A infecção osteoarticular é a complicação mais comum da brucelose (com taxas que variam de 10% a 85%), e causa artrite infecciosa em grandes articulações, espondilite, bursite, tenossinovite e osteomielite. 2 A infecção protética articular é uma complicação rara e menos relatada da brucelose. 3 4 Descrevemos um caso de infecção silenciosa por *Brucella* e apresentação incomum em uma prótese de quadril de 20 anos; também descrevemos seu tratamento e evolução, e revisamos a literatura disponível sobre infecção protética por *Brucella* . Segundo a literatura, este é o primeiro caso de infecção protética por *Brucella* descrito no Sudeste Asiático, e com maior tempo entre a cirurgia primária e o início da infecção.

## Relato de Caso

Uma paciente do sexo feminino, de 69 anos, operada havia 20 anos com prótese parcial unipolar de quadril por fratura deslocada do colo do fêmur, apresentou queixa de dor na parte superior da coxa ao carregar peso havia 3 meses e claudicação havia 1 ano. Ela não apresentava histórico de queda recente, trauma leve ou febre. A paciente era diabética e agricultora, e trabalhava com gado leiteiro. Ao exame, o membro acometido estava encurtado em 2 cm, e todos os movimentos do quadril, principalmente as rotações, eram dolorosos. O exame local não foi digno de nota. Não havia histórico sugestivo de infecção local ou sistêmica no passado recente ou logo após a cirurgia primária. As radiografias revelaram uma antiga prótese unipolar em monobloco cimentada *in situ* com subsidência da haste, colapso em varo e perfuração da cortical lateral ( Fig. 1 ). O exame de sangue revelou contagens totais e diferenciais de leucócitos, níveis de proteína C-reativa e velocidade de hemossedimentação normais. Uma artroplastia de quadril de revisão em estágio único foi planejada devido ao mau estado cardíaco da paciente.

**Fig. 1:** Radiografia pré-operatória em projeções anteroposterior e em perfil.

O quadril acometido foi exposto por meio da abordagem de Gibson modificada. O implante estava ligeiramente solto e, para surpresa dos autores, havia uma camada de material caseoso esbranquiçado em volta da junção osso-implante em nível trocantérico, o que levou à suspeita de infecção tubecular ou uma infecção similar. Uma osteotomia trocantérica estendida (OTE) foi feita até o nível da perfuração da cortical lateral da ponta da haste, e o implante e o cimento foram removidos. Amostras de tecido foram retiradas de cinco locais ao redor do quadril. Um teste intraoperatório de esterase leucocitária foi negativo. A ferida foi lavada com peróxido de hidrogênio, 3 litros de solução salina e betadina 0,3%, e fechada de forma temporária. Após o preparo de um novo campo cirúrgico, a ferida foi lavada mais uma vez com 3 litros de solução salina, e a cerclagem profilática foi feita 2 cm distal à perfuração. Uma haste longa, não cimentada, do tipo Wagner, de tamanho 20 mm x 240 mm, foi inserida. Considerando a preservação da cartilagem acetabular, a ausência de dor na virilha, a solicitação persistente do anestesista para encerrar o caso no menor tempo possível e a perda sanguínea devido ao mau estado cardíaco da paciente, foi realizada hemiartroplastia bipolar, e a OTE foi fixada com 3 fios de cerclagem ( Fig. 2 ). A ferida foi tratada com 2 g de vancomicina em pó em volta do quadril após o fechamento da cápsula.

**Fig. 2:** Radiografia obtida no período pós-operatório imediato em projeção anteroposterior.

Depois da cirurgia, a paciente recebeu cefuroxima sistêmica por cinco dias. O período pós-operatório foi digno de nota. A cultura de tecidos revelou o crescimento de *Brucella melitensis* . Após consulta com o infectologista, a paciente recebeu doxiciclina e rifampicina por via oral por seis semanas. A paciente continuou assintomática depois disso, e nenhum sinal de infecção local foi observado até o último acompanhamento em um ano.

## Revisão de Literatura

As bases de dados PubMed, PubMed Central (PMC) e Scopus foram pesquisadas usando-se as palavras-chave *Brucella* , *prosthetic joint infection* (infecção de prótese articular) e *septic hip* (quadril séptico). Selecionamos todos os resultados de estudos relacionados à infecção de próteses articulares por *Brucella* e avaliamos as referências desses estudos. Encontramos 30 artigos sobre infecção de prótese articular por *Brucella* , que incluíam 36 pacientes, 13 dos quais apresentavam infecção da prótese da articulação do quadril. A maioria dos pacientes provinha do sul da Europa e do Oriente Médio. Ao todo, 9 dos 13 (69,2%) pacientes tinham histórico de contato com animais de fazenda ou consumo de laticínios não pasteurizados. Infecção após viagem a países endêmicos foi relatada por 5 dos 36 (13,89%) pacientes. A média de tempo entre a colocação da prótese e a infecção foi de 51 meses. Os sintomas mais comuns se restringiam ao quadril e à coxa acometidos (11 pacientes; 84,6%), ao passo que 1 (7,7%) paciente apresentou apenas sintomas sistêmicos, e 1 (7,7%), sintomas locais e sistêmicos. O afrouxamento do implante foi observado em radiografias de 9 (69,2%) pacientes. O método de tratamento mais comum foi a artroplastia de quadril de revisão em dois estágios (7 pacientes; 53,8%). O desfecho foi relatado como bom em todos os casos.

## Discussão

O diagnóstico da infecção da prótese articular por *Brucella* , especialmente numa região não endêmica, é um desafio devido à sua raridade e apresentação geralmente insidiosa. 5 No caso aqui relatado, o tempo entre a primeira cirurgia e a infecção foi de 20 anos, o mais longo documentado até o momento. Além disso, nossa paciente apresentava sintomas locais leves, sem quaisquer sinais sistêmicos. Na literatura, 6 7 dois pacientes com infecção da prótese articular por *Brucella* tinham histórico de brucelose sistêmica (1 e 6 anos após a artroplastia, respectivamente) antes do desenvolvimento de sintomas locais. Este é um ponto importante na anamnese para o diagnóstico de infecção da prótese articular por *Brucella* . Além disso, é importante elucidar o histórico de contato com animais de fazenda ou consumo de laticínios não pasteurizados. Os testes sorológicos para anticorpos contra *Brucella* são seguros e confiáveis para o diagnóstico pré-operatório 8 , mas a cultura de tecidos obtidos no período intraoperatório continua sendo o método mais confiável. Não há diretrizes de tratamento específicas devido à ausência de estudos prospectivos, randomizados e em grande escala, com relato de bons desfechos após a artroplastia de revisão em um ou dois estágios. Pacientes sem qualquer afrouxamento do implante e sintomas locais leves foram tratados apenas com desbridamento ou até mesmo de forma conservadora, com bons resultados. O tratamento antimicrobiano geralmente consiste em doxiciclina e rifampicina com ou sem aminoglicosídeo, com duração de 6 semanas a 2 anos, dependendo da regressão dos sintomas. 9 10

Mais estudos prospectivos/multicêntricos com amostras com uma ampla variedade de origens étnicas contribuirão para o estabelecimento de diretrizes e protocolos de tratamento apropriados.
